# An introduction to the My Body is Fit and Fabulous at home (MyBFF@home): a community-based weight loss intervention study among Malaysian housewives

**DOI:** 10.1186/s12905-018-0589-x

**Published:** 2018-07-19

**Authors:** Noor Safiza Mohamad Nor, Rashidah Ambak, Tahir Aris

**Affiliations:** Institute for Public Health, National Institutes of Health, Ministry of Health Malaysia, Jalan Bangsar, 50590 Kuala Lumpur, Malaysia

**Keywords:** MyBFF@home, Weight loss, Intervention study, Housewives, Malaysia

## Abstract

**Background:**

The national prevalence of overweight and obesity in Malaysia has been increasing in the past 10 years and many efforts have been implemented by the Malaysian government to combat obesity problem among the Malaysian population. The aim of this paper was to describe the background of the My Body is Fit and Fabulous at home **(**MyBFF@home) study (Phase II).

**Discussion:**

The MyBFF@home (Phase II) was a quasi-experimental study and it was conducted among overweight and obese housewives living in the urban areas in Malaysia. In this phase, the study involved a weight loss intervention phase (6 months) and a weight loss maintenance phase (6 months). The intervention group received a standard weight loss intervention package and the control group received group seminars related to women’s health. Measurements of weight, height, waist circumference, body composition, fasting blood lipids, dietary intake, physical activity, health literacy, body pain and quality of life were conducted during the study. Overweight and obese housewives from 14 People’s Housing/Home Project (PHP) in Federal Territory of Kuala Lumpur (Klang Valley) were selected as control and intervention group (*N* = 328). Majority of the participants (76.1%) were from the low socioeconomic group. Data were analysed and presented according to the specific objectives and the needs for the particular topic in the present supplement report.

**Conclusion:**

MyBFF@home is the first and the largest community-based weight loss intervention study which was conducted among overweight and obese housewives in Malaysia. Findings of the study could be used by the policy makers and the researchers to enhance the obesity intervention programme among female adults in Malaysia.

## Background

The My Body is Fit and Fabulous at home **(**MyBFF@home) is a community-based weight loss intervention study targeting overweight and obese housewives in urban areas in Malaysia. The MyBFF@home is one of the projects under the MyBFF research framework, and it was initiated by the Malaysian government to combat obesity problem among the Malaysian population. In the past 10 years, the national prevalence of overweight and obesity in Malaysia has been increasing. Based on the definition of obesity from the World Health Organisation (WHO 1998; Body Mass Index (BMI > 30.0 kg/m^2^), the National Health and Morbidity Survey (NHMS) Malaysia 2006, 2011 and 2015 have shown that the obesity problem was more prevalent among women compared to men (Women:17.4, 17.6 and 20.6%; Men:10, 12.7 and 15.0%) [1–3]. Many efforts have been implemented by the Ministry of Health Malaysia which include the development of the dietary guidelines for Malaysia, healthy lifestyle campaigns, training programme and research activities. The aim of this article was to describe the background of the MyBFF@home mainly on the phase II of the study.

## Discussion

There were 2 phases of the MyBFF@home study which included Phase I: Development of the weight loss intervention package, and Phase II: Weight loss intervention. This article will describe the details of Phase II of the MyBFF@home study. The design of the MyBFF@home (Phase II) was a quasi-experimental study. It was conducted for 12 months which involved a weight loss intervention phase (6 months) and a weight loss maintenance phase (6 months). Participants in the Federal Territory of Kuala Lumpur (Klang Valley), Malaysia were chosen as samples of the MyBFF@home study due to a high prevalence of obesity among women (17.6%) and among the homemaker/housewives (20.9%) in this area [2]. Housewives from 14 People’s Home/Housing Project (PHP) in the Federal Territory of Kuala Lumpur were recruited at baseline from January 2014 until June 2014 and they were then categorised as control and intervention group. The intervention group received a standard weight loss intervention package (Table 1), which was developed by the research team members through a systematic process. The content of the weight loss intervention package and the diet counselling protocol were also revised based on the pre-test and feedback from the housewives during the in-depth interviews [4, 5].

**Table 1:** Components for intervention and control group

Components for weight loss intervention (intervention group)	
1.	Individual Diet Counselling (1-h, 4 times during the intervention phase and 2 times during weight maintenance phase) – Individual Diet Counselling by Dietitian/ Nutritionist using a standardised weight loss intervention protocol and education tools
2.	Standard regime for exercise/physical activity (Individual: 7 days/week and group exercise: 1×/month) Total: 60 min – brisk walking (30 mins) – pillow dumb bell exercise with 12 steps (30 mins)
3.	Reduced calorie diet – 1200–1500 kcal/day (dietary modification based on the individual Estimated Energy Requirement) – Education on food labeling, portion control and food substitution (low fat, and low sugar choices – recipes book and fliers)
4.	Self-monitoring tools – Pedometer (with 7 days memory) to monitor steps on daily basis – 3-day food diary for housewives (2-week days and 1 weekend) once a month (within the same week) – 3-day physical activity diary with MET calendar (2 weekdays and 1 weekend) - once a month (within the same week)
Components for health seminars (control group)	
1.	Self-monitoring tools – 3-day food diary for housewives (2-week days and 1 weekend) once a month (within the same week) – 3-day physical activity diary with MET calendar (2 weekdays and 1 weekend) - once a month (within the same week)
2.	Health seminars (30 min) Seminar 1 (Baseline) Relaxation technique Seminar 2 (Follow up 1) Grooming session Seminar 3 (Follow up 2) Parenting and social problems in the family life Seminar 4 (Follow up 3) Stress management Seminar 5 (Follow up 4) Pap smear Seminar 6 (Follow up 5) Breast Self-Examination (BSE) technique Seminar 7 (Follow up 6) Cancer related to women

The control group was also defined as a ‘delayed intervention group’, whereby the participants received group seminars (Table 1) and two self-monitoring tools (Food Diary and Physical Activity Diary). After 12 months, the participants in the control group were then invited to take part in the follow up weight loss intervention study for another 6 months. The methodology and study flow chart of the MyBFF@home (Phase I and Phase II) and the detailed baseline characteristics of the study participants were reported elsewhere [4, 6]. Meanwhile, the methods and findings for the follow up study (delayed intervention group) of the MyBFF@home are included in the present supplement report by Abdul Aziz NS.

### Geographical description of the study location

**Fig. 1:**
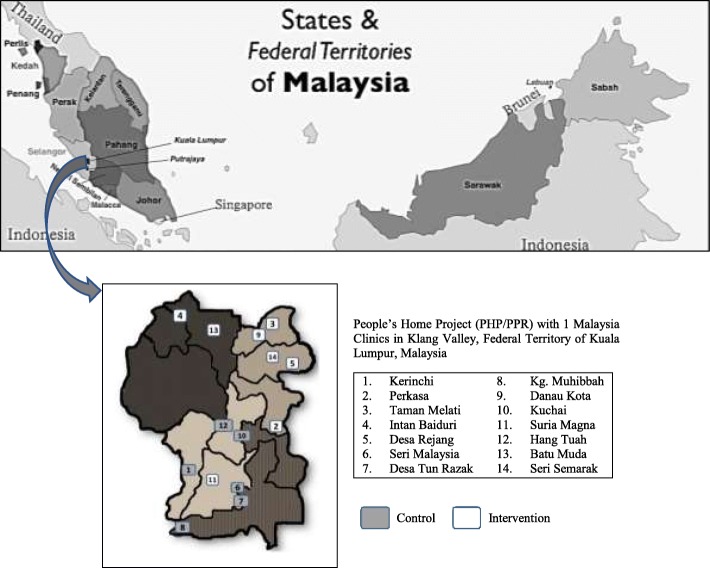
Study location of the MyBFF@home in Kuala Lumpur Federal Territory, Malaysia

Malaysia is a multi-ethic and multi-religious country in Southeast Asia consists of 13 states and three federal territories (Fig. 1). The estimated population for Malaysia in 2017 based on the Population and Housing Census is 32 million. The MyBFF@home study was conducted in the Federal Territory of Kuala Lumpur, which is located in the central area in Klang Valley, Malaysia. The estimated population in Klang Valley was 7.2 million, whereby the majority of ethnic group is Malay (50.6%), followed by Chinese (29.0%), Indian (11.7%), other ethnic group (0.7%) and non-Malaysian citizen (8.0%) [6]. There are many low-cost flats (People’s Housing Project - PHP) in Klang Valley, which were built by the Ministry of Urban Wellbeing, Housing and Local Government in order to support the housing needs of the low-income group (household monthly income less than RM 2500). In the MyBFF@home study, participants from 14 PHPs (attached with 1Malaysia Clinics) in the northern and southern area of Kuala Lumpur were selected as control and intervention group.

### Participant’s characteristics

In the MyBFF@home, housewives were defined as single/married/widowed female adults/homemakers (aged 18–59 years old) and have been staying at home (in the PHP) for at least 6 months prior to the recruitment. The inclusion criteria of participants in the MyBFF@home were housewives with the Body Mass Index (BMI) 25.0 to 39.9 kg/m^2^. Housewives who had physical disability, bed ridden, pregnant, history of medical conditions (diabetes, heart disease, renal dysfunction, severe hypertension) or currently on weight loss programme were excluded from the study. Housewives were identified through community representatives at the PHPs, while screening of the housewives was conducted by the Medical Assistants and the Public Health Nurses at the 1Malaysia Clinics. A total of 328 housewives from 14 PHPs have participated in the MyBFF@home study. Based on the baseline characteristics data, the monthly household income of the majority of the participants (76.1%) living in the PHPs were RM 2500 and in line with the criteria of the low socioeconomic group by the Ministry of Urban Wellbeing, Housing and Local Government [4].

In the intervention phase for 6 months, participants in the intervention group were given four (4) individual diet counselling sessions and group exercise sessions (brisk walking and dumb bell exercise) at 1 month(m), 2 m, 3 m and 6 m by trained health professionals (dietitians/nutritionists and physiotherapist). During the weight loss intervention phase, each participant in the intervention group was given 2 mini dumb bells (300 g each) and a pedometer to measure their daily steps. In addition, participants were also advised to engage in other moderate physical activities such as brisk walking, stairs climbing and house works up to 60 min per day. In the weight maintenance phase for another 6 months, participants in the intervention group were also given two (2) individual follow-up sessions with dietitians/nutritionists at 9 m and 12 m. The control group (delayed intervention group) received 7 group seminars related to women’s health and these seminars were given by the public health specialists and the health education officers who were not involved in the management of the intervention group. Measurements of weight, height, waist circumference, body composition, fasting blood lipids, dietary intake, physical activity, health literacy, body pain and quality of life were conducted during the study. The details of these measurements will be discussed in each topic of the supplement and the reporting of the findings utilised the TREND statement checklist for the intervention study [7]. Ethical approval for the MyBFF@home study was obtained from the Medical Research Ethic Committee (MREC) Malaysia (Research registration number: NMRR-13-726-16,391) and informed written consent was taken from all respondents at the beginning of the study.

## Conclusion

In this supplement report, the authors of individual topic have used the participants’ data from the MyBFF@home study. The results based on the data analysis were presented according to the objectives and the needs of the particular topic. Strengths and limitations of the study data will be also discussed by the different authors. To our knowledge, this is the first and the largest community-based weight loss intervention study among overweight and obese housewives in Malaysia. Findings of the MyBFF@home could be used by the policy makers and the researchers to enhance the obesity intervention programme among female adults in Malaysia.

## Abbreviations

BMI: Body Mass Index
MyBFF@home: My Body is Fit and Fabulous at home
NHMS: National Health and Morbidity Survey
PHP: People’s Home/Housing Project
WHO: World Health Organisation
